# Improving stock trading decisions based on pattern recognition using machine learning technology

**DOI:** 10.1371/journal.pone.0255558

**Published:** 2021-08-06

**Authors:** Yaohu Lin, Shancun Liu, Haijun Yang, Harris Wu, Bingbing Jiang

**Affiliations:** 1 School of Economics and Management, Beihang University, Beijing, China; 2 Key Laboratory of Complex System Analysis, Management and Decision (Beihang University), Ministry of Education, Beijing, China; 3 Beijing Advanced Innovation Center for Big Data and Brain Computing, Beihang University, Beijing, China; 4 Strome College of Business, Old Dominion University, Norfolk, Virginia, United States of America; 5 Software Engineering Center, Chinese Academy of Sciences, Beijing, China; The Bucharest University of Economic Studies, ROMANIA

## Abstract

PRML, a novel candlestick pattern recognition model using machine learning methods, is proposed to improve stock trading decisions. Four popular machine learning methods and 11 different features types are applied to all possible combinations of daily patterns to start the pattern recognition schedule. Different time windows from one to ten days are used to detect the prediction effect at different periods. An investment strategy is constructed according to the identified candlestick patterns and suitable time window. We deploy PRML for the forecast of all Chinese market stocks from Jan 1, 2000 until Oct 30, 2020. Among them, the data from Jan 1, 2000 to Dec 31, 2014 is used as the training data set, and the data set from Jan 1, 2015 to Oct 30, 2020 is used to verify the forecasting effect. Empirical results show that the two-day candlestick patterns after filtering have the best prediction effect when forecasting one day ahead; these patterns obtain an average annual return, an annual Sharpe ratio, and an information ratio as high as 36.73%, 0.81, and 2.37, respectively. After screening, three-day candlestick patterns also present a beneficial effect when forecasting one day ahead in that these patterns show stable characteristics. Two other popular machine learning methods, multilayer perceptron network and long short-term memory neural networks, are applied to the pattern recognition framework to evaluate the dependency of the prediction model. A transaction cost of 0.2% is considered on the two-day patterns predicting one day ahead, thus confirming the profitability. Empirical results show that applying different machine learning methods to two-day and three-day patterns for one-day-ahead forecasts can be profitable.

## 1. Introduction

Analyzing and forecasting the stock market is notoriously tricky due to the high degree of noise [1] and semi-strong form of market efficiency [2], which is generally accepted. A reasonably accurate prediction may raise the potential of yielding benefits and hedging against market risks. However, financial economists often question the existence of opportunities for profitable predictions [3].

Technical analysis, also called candlestick charting, is one of the most common traditional analysis methods to predict the financial market. By utilizing open-high-low-close prices in chronological order, candlestick charting can reflect not only the changing balance between supply and demand [4] but also the sentiment of the investors in the market [5]. Bulkowski described the known 103 patterns in natural language [6], and then comprehensive formal specifications of the known candlestick patterns were proposed [7]. In recent years, technical analysis has been proven to be effective in stock market analysis. For example, Caginalp and Laurent tested the predictive capability of candlestick patterns and found that applying candlestick trading strategies on daily stock returns in S&P 500 stocks can result in profits [8]. Goo et al. found that many one-day candlestick and reversal patterns can help investors earn significant returns by following candlestick trading strategies [9]. Moreover, the profitability of candlestick trading strategies was further confirmed [10, 11]. More complex candlestick patterns have been used in the latest research. The predictive power of 5 two-day reversal patterns was examined [12], and 4 pairs of two-day patterns were studied [13]. Lu et al. studied the profitability of 8 three-day reversal patterns under trend conditions and different holding strategies [11].

Although these studies have shown that using the candlestick pattern strategy can be profitable, dissenting voices have emerged in academia. Fock et al. found no evidence of the predictive ability of candlestick patterns alone or in combination with other common technical indicators in the DAX stock index contract and the Bund interest rate future [14]. Duvingage et al. tested the intraday predictive power of Japanese candlesticks at the 5-minute interval on the 30 constituents of the DJIA index and concluded that candlestick trading strategies do not improve investment performance [15]. These conflicting conclusions about candlestick patterns prompt us to investigate further.

Artificial intelligence has recently been applied to address the chaotic and randomness time series data [16, 17]. The intense computational use of intelligent predictive models has commonly been studied under machine learning [18]. Compared to the more traditional models, machine learning models provide more flexibility [19], do not require distributional assumptions, and can easily combine individual classifiers to reduce variance [20]. Many machine techniques have already been applied to forecast the stock market [20–36]. For example, logistic regression (LR) and neural networks (NNs) [27, 29, 30, 36], deep neural networks (DNNs) [22], decision trees (DTs) [22, 25], support vector machines (SVMs) [24, 26, 28] or support vector regression (SVR) [21], k-nearest neighbors (KNN) [23, 33], random forests (RFs) [22], long short-term memory networks (LSTMs) [1, 31, 34] and restricted Boltzmann machines (RBMs) [32] have been used to predict stock market movements. In comparing multiple machine learning methods, Fischer et al. (2018) deploy LSTM networks for predicting out-of-sample directional movements for the constituent stocks of the S&P 500 from 1992 until 2015. They find LSTM networks to outperform RF, DNN and LR. Patel et al. (2015) compare the performance of four models (namely, ANN, SVM, RF, and naïve-Bayes) with respect to the following indexes and companies on the Indian stock market: CNX Nifty, S&P BSE Sensex, Infosys Ltd. and Reliance Industries [35]. Brownstone (1996) used a neural network to predict daily closing prices for five days and twenty-five days of the FTSE 100 Share Index in the UK and used multiple linear regression to compare the prediction results [30]. Krauss et al. (2017) implemented and analyzed the one-day effectiveness of deep neural networks (DNNs), gradient-boosted-trees (GBTs), and random forests (RFs) on all stocks of the S&P 500 from 1992 to 2015, and then the trading signals were generated based on the forecast probability. These techniques sort all stocks over the cross-section *k* probability in descending order. Five different investment strategies are generated by going long the top *k* stocks and short the bottom K stocks, with k∈{10,50,100,150,200}. These techniques achieve the best performance when *k* = 10, and RF outperforms GBT and DNN [22]. Profitable patterns may be discovered based on more recent returns and daily data. Qiu et al. (2020) established an LSTM with a wavelet forecasting framework to predict the opening prices of stocks [34].

The existing research shows that machine learning methods can effectively predict the direction of the financial market. However, the use of machine learning technology for candlestick pattern recognition is still less prevalent. Moreover, traditional research on candlestick patterns focuses mainly on a limited number of patterns [9–15]. The latest artificial intelligence technology prompts us to consider applying pattern recognition to decision-making in the stock market. Since different machine learning methods perform differently in different scenarios [1, 22, 30, 35], a prediction framework that can adapt to different machine learning methods will greatly improve the usability of the model.

The remainder of this paper is organized as follows: Section 2 outlines the design of this paper. A pattern recognition prediction framework with four popular machine learning methods is designed. Then two other popular machine learning methods are used to replace the four machine learning methods to evaluate the dependency of the prediction framework. Section 3 presents the empirical results. Section 4 concludes the paper.

## 2. Methodology

**Fig 1:**
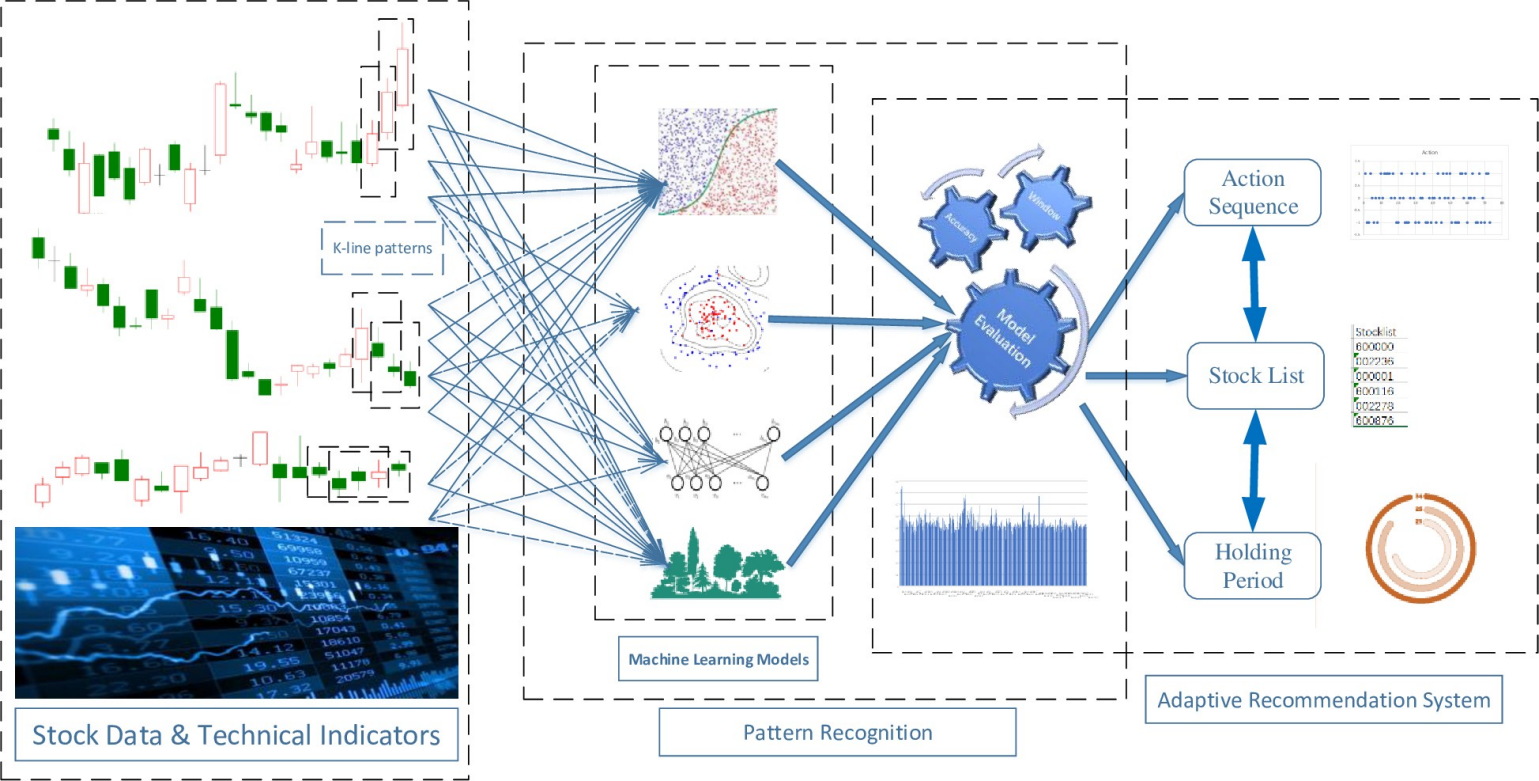
Overview of the PRML model.

This paper attempts to develop PRML, a Pattern Recognition method based on Machine Learning methods, to improve stock trading decisions, as shown in **Fig 1**. First, 13 forms of one-day patterns are constructed and classified, and then the corresponding technical indicators and location information are calculated. Then, the pattern-building phase starts; this phase generates all possible combinations of candlestick patterns. For example, the two-day patterns are composed of two consecutive one-day patterns; therefore, there are 13*13 possible combinations. All the pattern information, including the technical indicators and location information, is put into the machine learning models, which test the prediction accuracy of each pattern during different periods. The pattern recognition stage retains only those patterns whose accuracy exceeds the threshold value. Finally, the adaptive recommendation schedule gives corresponding stock prediction actions based on the evaluated results.

**Fig 2:**
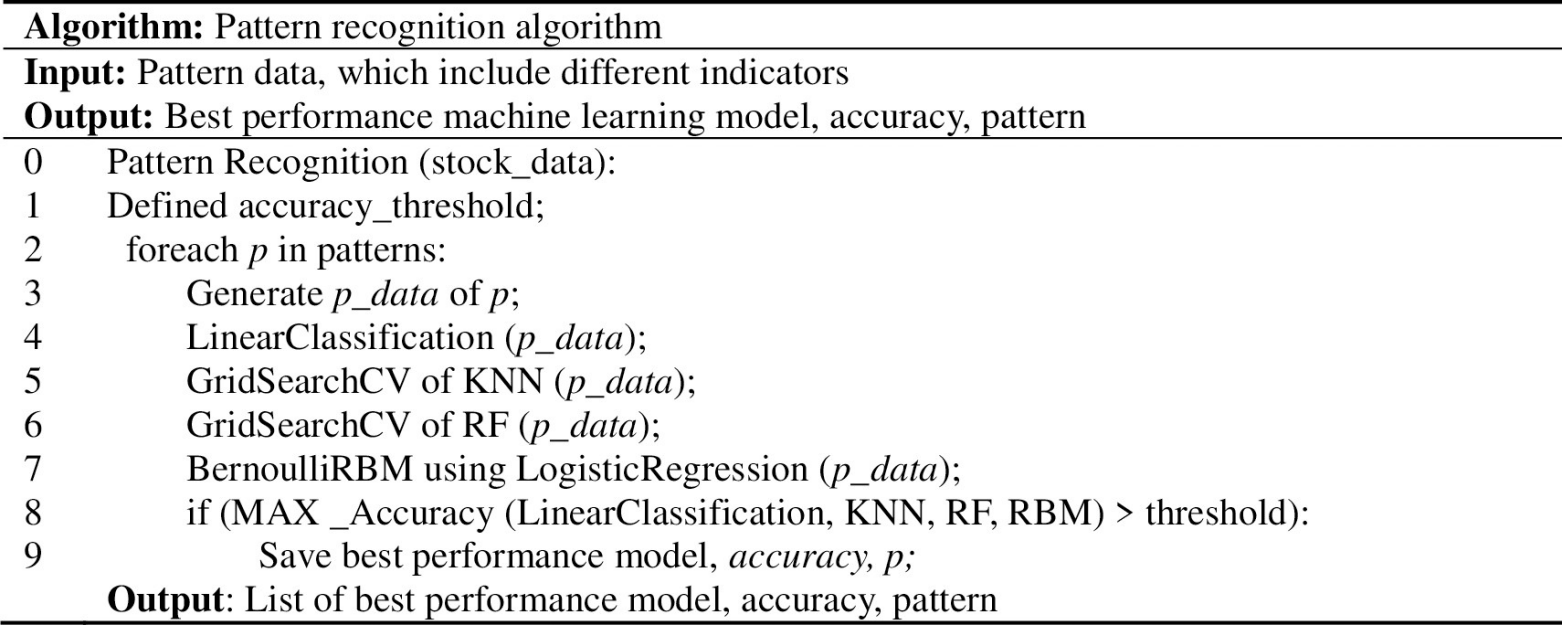
Main pattern recognition algorithm.

Based on the identified patterns, the daily portfolio is dynamically built. The main pattern recognition process is shown in **Fig 2**.

### 2.1 Candlestick patterns

**Fig 3:**
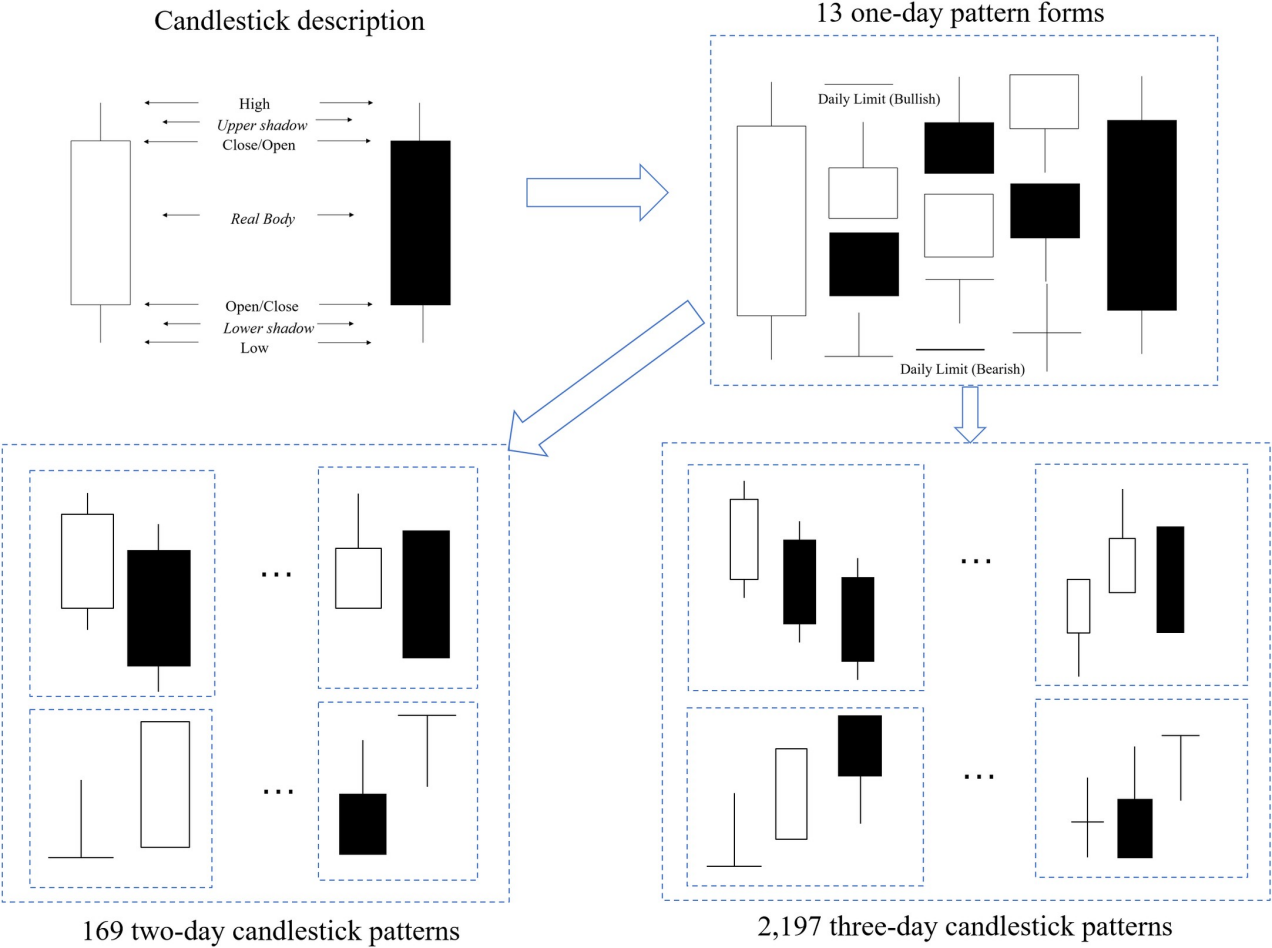
Description of a candlestick and candlestick patterns.

A candlestick, also called a K-line, consists of four basic elements: the opening price, high price, low price and closing price, as shown in **Fig 3**. For simplicity, these elements are often denoted as open, high, low, and close. According to the different values of open, high, low and close, one-day patterns can have 13 different forms, as shown in **Fig 3**.

#### 2.1.1 Definitions

The definitions and functions used to describe the rules for classifying daily candlestick patterns are listed as follows:

*2*.*1*.*1*.*1 Definition 1 (Candlestick)*. A candlestick *k =* (*o*_*t*_, *h*_*t*_, *l*_*t*_, *c*_*t*_) is a tuple that consists of four basic prices of a stock at time *t*. A candlestick *k* is a basic element in identifying the candlestick pattern recognition. The *o*_*t*_, *h*_*t*_, *l*_*t*_ and *c*_*t*_ represent the opening price, high price, low price and closing price at time *t*, respectively. Additionally, *k*_*i*_ *=* (*o*_*it*_, *h*_*it*_, *l*_*it*_, *c*_*it*_) denotes the *i*^*th*^ candlestick at time *t*.

*2*.*1*.*1*.*2 Definition 2 (Candlestick time series)*. A candlestick time series *T*_*n*_ = {*k*_1_, *k*_2_, …, *k*_*n*_} is a sequence of candlesticks of a stock; the sequence consists of n candlesticks from day 1 to day n. Additionally, *T*_*in*_ = {*k*_*i*1_, *k*_*i*2_, …, *k*_*in*_} denotes the *i*^*th*^ stock sequence.

*2*.*1*.*1*.*3 Definition 3 (Candlestick relative position)*. The candlestick relative position is defined as:

$$loc=\{\begin{array}{cc} BC_{h} & where\;h_{t}>h_{t-1},l_{t}<l_{t-1},c_{t}>c_{t-1}\\ BC_{l} & where\;h_{t}>h_{t-1},l_{t}<l_{t-1},c_{t}<c_{t-1}\\ BH_{h} & where\;h_{t}>h_{t-1},l_{t}>l_{t-1},c_{t}>c_{t-1}\\ BH_{l} & where\;h_{t}>h_{t-1},l_{t}>l_{t-1},c_{t}<c_{t-1}\\ BL_{h} & where\;h_{t}<h_{t-1},l_{t}<l_{t-1},c_{t}>c_{t-1}\\ BL_{l} & where\;h_{t}<h_{t-1},l_{t}<l_{t-1},c_{t}<c_{t-1}\\ BM_{h} & where\;h_{t}<h_{t-1},l_{t}>l_{t-1},c_{t}>c_{t-1}\\ BM_{l} & where\;h_{t}<h_{t-1},l_{t}>l_{t-1},c_{t}<c_{t-1} \end{array}$$

*loc*_*i*_ denotes the relative position of the *i*^*th*^ candlestick.

*2*.*1*.*1*.*4 Definition 4 (Candlestick relative position series)*. *LOC*_*n*_ = {*loc*_1_, *loc*_2_,…, *loc*_*n*_} is a sequence of relative position information of a stock; the sequence consists of n relative position information from day 1 to day n.

*2*.*1*.*1*.*5 Definition 5 (Candlestick pattern)*. A candlestick pattern or K-line pattern *p*_*j*_ = {*T*_*j*_, *Loc*_*j*_} is a subsequence of consecutive candlesticks; this subsequence consists of two parts: a sequence of candlesticks and a corresponding location sequence. For example, a two-day pattern can be defined as *p*_2_ = {*T*_2_, *Loc*_2_} and *T*_2_ = {*k*_1_, *k*_2_}, *Loc*_2_ = {*loc*_1_, *loc*_2_}.

### 2.2 Technical indicators

Technical indicators are mathematical calculations based on price and volume. By analyzing historical data, technical analysts use indicators to predict the future trend of the financial market [37, 38], and these indicators can potentially affect stock price prediction [39, 40]. Following Zhou et al. (2019) and Bao et al. (2017), we use feature sets containing several indicators that are commonly used in the technical analysis [3, 31]. The most prevalent indicators are defined below:

- A moving average (MA) is a calculation to analyze data points by creating a series of averages of different subsets of the full data set. The calculation formula is:

$$MA\left(t\right)=\frac{1}{m}\overset{m-1}{\underset{i=0}{\sum }}c_{t-i}$$ (1)

where *m* refers to the time interval and *c* is the closing price.

- An exponential moving average (EMA) is a first-order infinite impulse response filter that applies weighting factors, which decrease exponentially. The calculation formula is:
$$EMA\left(t\right)=\frac{2}{n+1}c_{t}+\frac{n-1}{n+1}EMA\left(t-1\right)$$ (2)
  Where *n* refers to the time interval.
- The volume rate of change (ROC) shows the changes in volume. The calculation formula is:
$$ROC\left(t\right)=\frac{v_{t}}{\frac{1}{x}\sum_{i=0}^{x}v_{t-i}}$$ (3)
  Where *x* refers to the time interval and *v*_*t*_ refers to the volume at time *t*.
- The commodity channel index (CCI) is designed to detect the beginning and the ending market trends and measures a security’s variation from the statistical mean. The calculation formula is:
$$CCI\left(t\right)=\frac{\frac{h_{t}+l_{t}+c_{t}}{3}-MA\left(n\right)}{0.015*\frac{1}{n}\sum_{i-n}^{n}MA\left(i\right)-c_{i}}$$ (4)
- Momentum (MOM) measures the acceleration and deceleration of prices. The calculation formula is:
$$\text{MOM}\left(\text{t}\right)=c_{t}-c_{t-n}$$ (5)
- The Chaikin A/D line (AD) measures the accumulation-distribution line and is calculated as follows:
$$Clv\left(t\right)=\frac{2*c_{t}-h_{t}-c_{t}}{h-l_{t}}$$ (6)
$$AD\left(t\right)=AD\left(t-1\right)+v_{t}*Clv\left(t\right)$$ (7)
- On balance volume (OBV) is a cumulative total of the up and down volume. The calculation logic of *OBV* is as follows:
  if *c*_*t*_ > *c*_*t*-1_
$$OBV\left(t\right)=OBV\left(t-1\right)+v_{t}$$ (8)
  if *c*_*t*_ < *c*_*t*-1_
$$OBV\left(t\right)=OBV\left(t-1\right)-v_{t}$$ (9)
  else
OBV(t)=OBV(t−1) (10)
  where *v* refers to the volume.
- The true range (TRANGE) is a base calculation that is used to determine the normal trading range of a stock or commodity. The calculation formula is:
$$TR\left(t\right)=\max\left(H_{t},\;C_{t-1}\right)-min\left(L_{t},\;C_{t-1}\right)$$ (11)
- The average true range (ATR) is a moving average of the true range. The calculation formula is:
$$ATR\left(t\right)=\frac{1}{n}\overset{n}{\underset{i=1}{\sum }}TR\left(t-i+1\right)$$ (12)

### 2.3 Pattern generation & generation of training and testing sets

We begin to extract more complex N-day daily patterns based on the one-day candlestick patterns. First, the one-day candlestick pattern classification is generated on the basis price of open, high, low, and close. A total of 13 one-day patterns are obtained after considering all the circumstances. Second, the values of nine technical indicators, shape and relative location information are calculated. The research in this paper focuses on short-term effects; 5 days or 10 days as the parameters of indicators are choosen. The parameters of 5 days for *MA* and 10 days for *EMA*, *CCI* and *ATR* are used for testing. All the characteristics of the one-day patterns are obtained through these two processes. Finally, the one-day candlestick patterns are combined into more complex patterns for each stock according to different time windows. A total of 169 two-day candlestick patterns are combined based on the 13 one-day candlestick patterns. Time must be continuous in this pattern combination process.

After all the patterns and features are ready, we begin to prepare the training sets and testing sets. The entire data set are divided into two parts: the machine learning data set from Jan 1, 2000 to Dec 31, 2014 and the forecasting set from Jan 1, 2015 to Oct 30, 2020. Of the machine learning data set, 80% is used as training subsets, and 20% is used as testing subsets. First of all, the data of the training subset is used to fit the parameters in the machine learning model. Next, the machine learning model that fits the parameters is predicted in the testing subset, and the predicted result is compared with the real value to get the accuracy rate. The testing subset is the validation set. The corresponding result is the next N-day’s direction of the close price; N is from 1 to 10.

### 2.4 Prediction models

The inference engine is introduced in this phase. We use four machine learning models, logistic regression (LR), k-nearest neighbors (KNN), random forest (RF), and restricted Boltzmann machine (RBM), to predict the direction of the close price. The parameters used in the four machine learning methods are shown as **Table 1**.

**Table 1:** Parameters of the four machine learning models used in prediction schedule.

MLs	Parameters
LR	Regularized = *L2*, solver_parameter = *warn*, *C = 1*.*0*, *iteration = 100*, *criteria = 0*.*0001*
KNN	n_neighbors = range(1,10), weights = [’uniform’,’distance’], algorithm = [’auto’,’ball_tree’,’kd_tree’,’brute’], leaf_size = range(1,2), optimizer = *GridSearchCV*, cv = 10
RBM	learning_rate = 0.06, iteration = 10, *C* = 6000, components = 100
RF	n_estimators = range(10,100,5), criterion = [gini, entropy], min_samples_leaf = [2, 4, 6,50], max_depth = range(1,10), optimizer = *GridSearchCV*, cv = 10

#### 2.4.1 Logistic regression (LR)

Logistic regression is the most basic machine learning algorithm. The logistic regression model returns an equation that determines the relationship between the independent variables and the dependent variable. The model calculates linear functions and then converts the result into a probability. Finally, the model converts the probability into a label.

In the empirical stage, we use *L2* as a regularized parameter and specify *warn* as the solver parameter that determines our optimization method for the logistic regression loss function. In terms of the termination parameters of the algorithm, we set the maximum number of iterations that are taken for the solvers to converge to *100* and set the tolerance for the stopping criteria parameter to *0*.*0001*.

#### 2.4.2 K-nearest neighbors (KNN)

K-nearest neighbors (KNN) is another machine learning algorithm. The k-NN algorithm looks for ‘k’ nearest records within the training data set and uses most of the classes of the identified neighbors for classifying. Subha used k-NN to classify the stock index movement [23], and Zhang et al. used ensemble empirical mode decomposition (EEMD) and a multidimensional k-nearest neighbor model (MKNN) to forecast the closing price and high price of the stocks [33].

In the experimental stage, we obtain the best performance from the grid search algorithm, which sets different neighbors, leaves, and weights. Different parameter combinations produce different clustering effects.

#### 2.4.3 Restricted Boltzmann machine (RBM)

A restricted Boltzmann machine (RBM) is a generative stochastic artificial neural network that can learn a probability distribution over its input sets. Recently, due to their powerful representation, RBMs have been used as generative models of many types of data, including text, images and speech. Liang et al. used an RBM to predict short-term stock market trends [32].

In the empirical stage, we connected a logistic regression to the output of the RBM for classification. Different parameter combinations may produce different classification effects; therefore, we set *10* iterations and *100* components to improve training results.

#### 2.4.4 Random forest (RF)

The following brief description of random forests follows Breiman (2001). Random forests are a combination of tree predictors such that each tree depends on the values of a random vector sampled independently and with the same distribution for all trees in the forest. Random forests (RFs) are nonparametric and nonlinear classification and regression algorithms [41]. Random forests not only use a subset of the training set but also select only a subset of the feature set when the tree is established in the decision tree. In the training stage, RF repeats *n* times to select a random sample with replacement of the training set and selects *k* features randomly to build a decision tree. Then repeat the above steps *T*_*num*_ times to build *T*_*num*_ decision trees. After each tree decision, the final result is confirmed by voting. Booth (2014) used RFs to construct an automated trading mechanism [42].

In the empirical stage, RF repeats 5000 times to select a random sample with replacement of the training set and selects $\sqrt{K}$ features, where *K* is the number of the input features. There are three tuning parameters: the number of trees *T*_*num*_, their maximum depth *T*_*depth*_, and the minimum number of samples required to be at a leaf node *T*_*sample*_. We set these parameters as *T*_*num*_ ∈ [10,100], *T*_*depth*_ ∈ [1,10] and *T*_*sample*_ ∈ {2, 4, 6, 50} into a relatively large selectable range and use the grid search algorithm to find the optimal performance.

### 2.5 Two other testing machine learning models

#### 2.5.1 Multilayer perceptron network (MLP)

A multilayer perceptron (MLP) is a class of feedforward artificial neural network (ANN). A MLP consists of an input layer, one or more hidden layers and an output layer. The following brief description of MLP follows Moghaddam et al. (2016) [36]. The input layer matches the feature space, and the output layer matches the output space, which may be a classification or regression layer. In the network, each neuron in the previous layer is fully connected with all neurons in the subsequent layer and represents a certain weight. Each non-output layer of the net has a bias unit, serving as an activation threshold for the neurons in the subsequent layer. In this study, the most common three-layer MLP model is constructed based on experience. In order to improve the generalization of the model, the number of inputs is set to 64, which is greater than the number of features. Finally, a three-layer MLP network, including an input layer with 64 nodes, a hidden layer with 64 neurons and an output layer with 1 neuron is developed. The *ReLU* activation, 0.1 leaning rate, 20 epochs, 128 batch sizes and an *RMSProp* optimizer for the objective function of *binary_crossentropy* are used in the learning phase.

#### 2.5.2 Long short-term memory neural networks (LSTMs)

Long short-term memory neural networks (LSTMs) are one of the most common forms of recurrent neural networks (RNNs), which are a type of deep neural network architecture. This description of LSTMs follows the description of Fischer et al. (2018), Bao et al. (2017) and Qiu et al. (2020) [1, 31, 34]. The LSTM consists of a set of memory cells that replace the hidden layer neurons of the RNN. The memory cell consists of three components: the input gate, the output gate, and the forget gate. The gates control the interactions between neighboring memory cells and the memory cell itself. The input gate controls the input state, while the output gate controls the output state, which is the input of other memory cells. The forget gate can choose to remember or forget its previous state. In this study, a common three-layer LSTM model is constructed based on experience. The number of inputs is set to 64 to improve the generalization. Finally, a three-layer LSTM network that includes 64 neurons input and an *Adam* optimizer for the objective function of *binary_crossentropy*, a middle layer with 64 neurons and an output layer with 1 neuron is developed; 10 *epochs* are constructed to increase the stability. A total of 56,129 parameters are generated by the LSTM in the prediction process.

### 2.6 Model evaluation

The machine learning models are used to forecast stock fluctuations. To evaluate the performance of the prediction models, the common evaluation criterion of *Accuracy* is used in this study. *Accuracy* is used to evaluate the overall classification ability of the model. The formula is as follows:

$$Accuracy=\frac{TP+TN}{TP+TN+FP+FN}$$ (13)

where *TP (true positive)* indicates that both the model prediction and the real sample are true; *TN (true negative)* indicates that both the model prediction and the real sample are false; *FP (false positive)* indicates that the model prediction is true but that the real sample is false; and *FN (false negative)* indicates that the model prediction is false while the real sample is true. In the empirical stage, patterns are selected into the strategy pool when the model’s accuracy rate is higher than 55%.

In the dependence testing stage, the *F-measure* is also introduced to evaluate the performance of MLP and LSTM. Following Patel et al. (2015) and Zhou et al (2019), the *F-measure* evaluation method and the additional metrics are defined as follows [3, 35]:

$$Precision=\frac{TP}{TP+FP}$$ (14)

$$Recall=\frac{TP}{TP+FN}$$ (15)

$$F-measure=\frac{2*Precision*Recall}{Precision+Recall}$$ (16)

*Recall* is also called sensitivity or the true positive rate. The *F-measure* is an increasing function when *Precision* and *Recall* are equally important.

Four statistical measures are chosen to evaluate the trading performances, including Sharpe Ratio, Maximum Drawdown, Average Annual Return and Information Ratio. These four measures are defined as follows:

$$SharpeRatio=\frac{\mu (R_{t})-r_{f}}{\sigma (R_{t})}$$ (17)

Where *R*_*t*_ is the cumulative return until date *t*, *μ(R*_*t*_*)* and *σ(R*_*t*_*)* are the corresponding mean and standard deviation of the return *R*_*t*.,_ *r*_*f*_ *is the risk-free return*.

$$MaximumDrawdown=\underset{\tau \in \left(0,T\right)}{\max}\left(\underset{t\in \left(0,\tau \right)}{\max}\left(R_{t}-R_{\tau }\right)\right)$$ (18)

Where *T* denotes a period.

$$AverageAnnualReturn={\left(\overset{N}{\underset{i=1}{\prod }}(1+r_{i})\right)}^{\frac{1}{N}}-1$$ (19)

Where *r*_*i*_ denotes the return of year *i*.

$$InformationRatio=\frac{R_{t}-R_{b}}{\sigma_{t}}$$ (20)

Where *R*_*b*_ is the benchmark return.

The Sharpe Ratio depicts the risk-adjusted return, the Maximum Drawdown denotes the largest cumulative loss over the period of investment and the Information Ratio measures the portfolio returns beyond the returns of a benchmark.

### 2.7 Investment strategy

**Fig 4:**
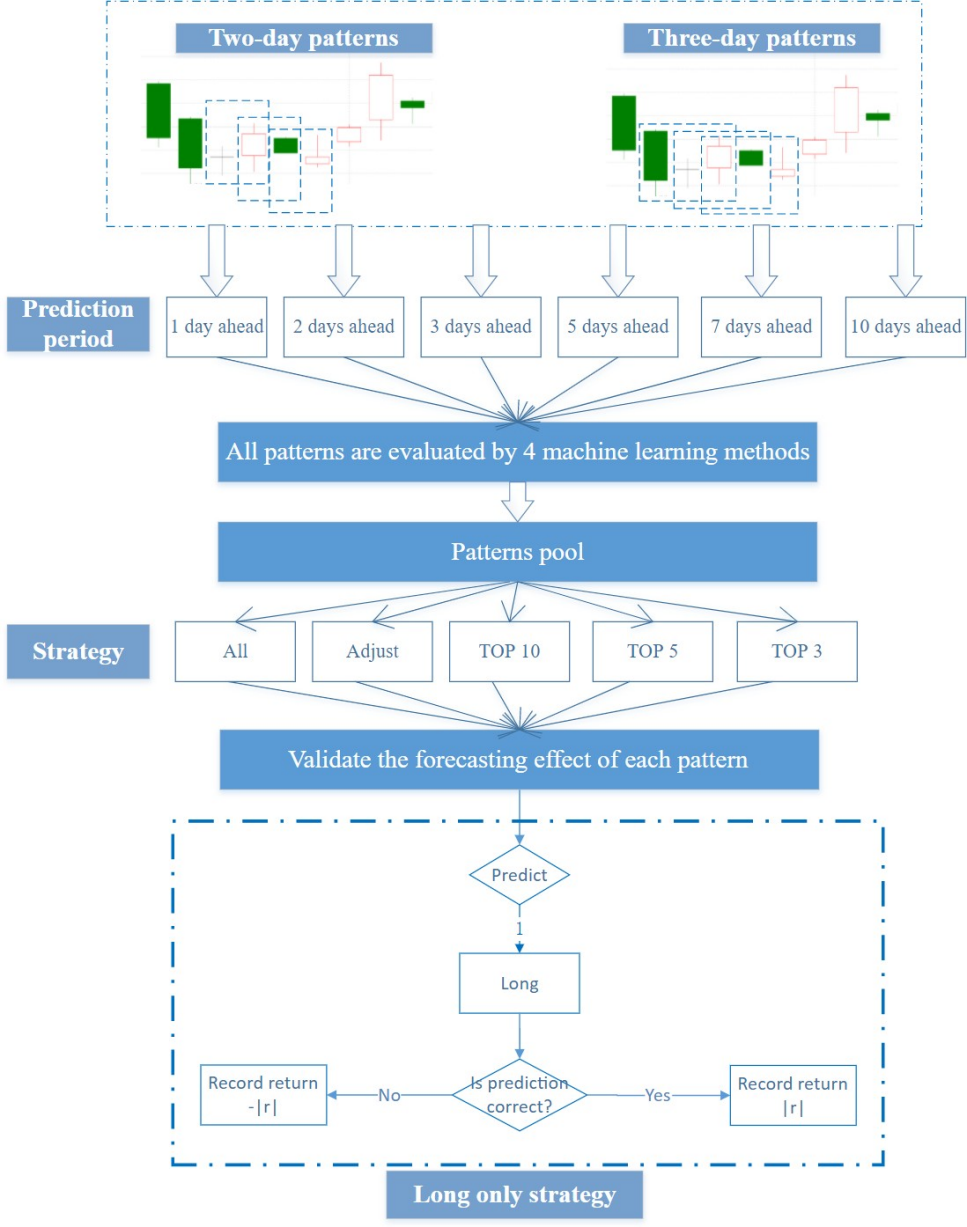
Flowchart of investment strategy construction for two-day patterns and three-day patterns.

Based on the above evaluation criteria, the investment strategy is constructed. According to the actual situation of the Chinese stock market, short selling is limited to some stocks with securities margin trading. Therefore, we consider only long selling and build the corresponding investment strategy with different time windows from 1 day to 10 days. This article assumes that we will invest at the close price at time *t* and will be clear at the close price at time *t+N*, where *N* is from1 to 10. Suppose that we have initial capital *M* and the current stock can be bought without affecting price fluctuations; then, the specific construction steps of the equal-weight investment strategy are as follows:

First, all the possible two-day and three-day candlestick patterns of all stocks traded on the Chinese stock market are checked at time *t*. Next, the machine learning methods are used to predict the rise or fall of *t+N* based on the above evaluation model. Only the best-performing of the machine learning methods and forecasting results will be saved after comparing the prediction results of these machine learning models. If the predicted result is long and consistent with the real result, the t+*N* profit is recorded. If the prediction is wrong, the negative profit of *t+N* is recorded as a loss value. Furthermore, it would do nothing if the predicted result is short. Then, the above steps are repeated to calculate *t+1*, *t+2*, etc. Finally, the recommendation stage for investment is carried out. The specific patterns where accuracy exceeds the threshold are screened out in the training and testing stage. The daily investment portfolio is adaptively built according to the filtered patterns pools. Five different strategy pools are used to form 5 comparable strategies: the *All*, *Adjust*, *TOP10*, *TOP5* and *TOP3* candlestick pattern pool. The *All* baseline candlestick pattern pool contains all the candlestick patterns whose accuracy is greater than 55% in the testing stage. After excluding from the rule pool the patterns that appear fewer than *1000* times in the machine learning set, from the *All* candlestick pattern pool, we obtain the *Adjust* patterns pool. The 10 most accurate patterns in the adjust pool were used to form the *TOP10* candlestick patterns pool; the 5 most accurate patterns in the adjust pool were used to form the *TOP5* candlestick patterns pool; and the 3 most accurate patterns in the adjust pool were used to form the *TOP3* candlestick patterns pool. The investment flowchart is shown as **Fig 4**.

In this equal-weight investment strategy, there is *M/N* capital each day. Suppose there are *P*_*i*_ patterns in the *i*-*th* investment strategy, then each pattern has $\frac{M}{N}*\frac{1}{P_{i}}$ capital for investment. Suppose there are *K* stocks in pattern *p*_*i*_ at time *t*, then a total amount of $\frac{M}{N}*\frac{1}{P_{i}}*\frac{1}{K_{i}}$ capital will be invested on each stock. Finally, the average daily return of the portfolio is calculated in the forecasting stage to test prediction performance.

## 3. Empirical results

### 3.1 Data and training environment

In this study, we use the daily data on the Chinese stock market from the period of Jan 1, 2000 to Oct 30, 2020; a list of 9,745,597 rows of original data is used in our study.

Stock data are collected from CCER, a local data provider in China. First, a data cleaning phase schedule is carried out to guarantee the validity of the training data. We remove the daily data for a given stock if the trading volume is zero, which is a sign of stopped trading due to, e.g., company reorganization. Rows that contain one or more missing values out of range are removed from the database.

Then, feature information for each stock is generated on each day *t*. According to the definition of the candlestick chart, the feature of *Shape* is labeled. The feature of *Loc* is generated by using the definition of candlestick relative position. The other 9 feature values are calculated by indicator formulas (1)-(12). Therefore, the daily data contains these 11 features for each stock. To ensure effectiveness, three rounds of training are carried out. We randomly choose 5,000 rows of daily stock data for each of the patterns from the database. To ensure the balance of classification during training, for each intraday pattern, we choose half of the training data with rising prices (closing price lower than next N-day’s) and half of the training data with falling prices. Finally, the average accuracy is obtained based on the three rounds of results.

As a result, a list of 5,445,915 rows of the two-day patterns data with 26 data columns and 5,420,650 rows of the three-day patterns data with 38 data columns is generated. The *Date* and *Result return* information used for investment return calculation will not be used in machine training. Therefore, 22 distinct features will be used as input data for two-day candlestick patterns recognition and 33 distinct features will be used as input data for three-day candlestick patterns recognition. Regardless of the two-day or three-day patterns, the *Result direction* data is used for training and evaluation of machine learning results. The features of the two-day patterns are composed of the features of the first day, the next day, and the result features. In addition to the features of the two-day patterns, the three-day patterns also include the features of the third day. The data sample which used in the machine learning models is shown as **Table 2.**

**Table 2:** Data samples used in the machine learning models.

First day features											
Date	Shape	Loc	MA5	EMA10	ROC(1)	CCI10	MOM10	AD	OBV	TR	ATR10
20081119	12	6	10.59	10.26	0.75	82.99	2.11	1473163	34274056	1	0.89
The next day features											
Date	Shape	Loc	MA5	EMA10	ROC(1)	CCI10	MOM10	AD	OBV	TR	ATR10
20081120	11	7	10.64	10.31	1.12	65.39	2.09	1161726	33662716	0.57	0.86
Third day features											
Date	Shape	Loc	MA5	EMA10	ROC(1)	CCI10	MOM10	AD	OBV	TR	ATR10
20081121	11	5	10.56	10.29	0.89	6.77	1.44	1115630	33116148	0.83	0.85
Result features											
Result direction				Result return							
0				-4.4							

### 3.2 Model comparison and evaluation

**Fig 5:**
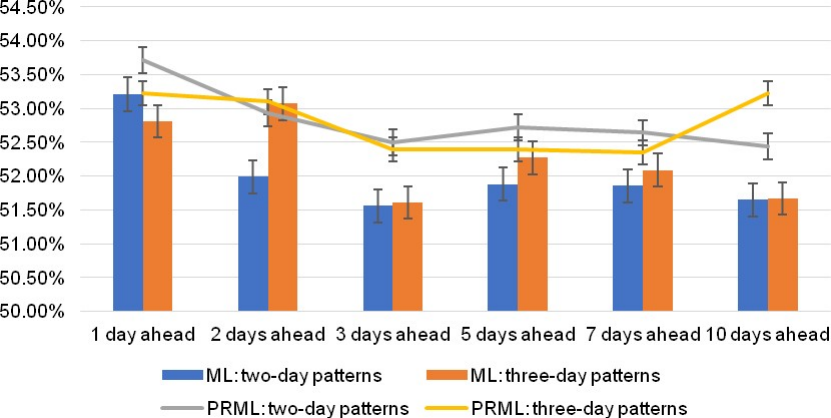
Accuracy overviews of PRML and pure machine learning models.

Four complete predictions are made in this study. First, all two-day candlestick patterns and three-day candlestick patterns data are put into the four machine models for prediction without distinguishing patterns. The histograms in **Fig 5** show the forecast accuracy in the training sets for 1, 2, 3, 5, 7, and 10 days ahead. Then, a prediction method for a basic segmentation pattern is performed. All possible combinations of different patterns for two-day and three-day patterns are put into four machine learning models to make predictions and record the best machine learning method and accuracy rate for each pattern. During training, patterns that appear fewer than 100 times are discarded. Three full-round calculating works have been done and the error bars are calculated based on the standard deviation of the three groups of calculation results. In the forecasts 2, 3, 5, 7 and 10 days ahead, the pure ML methods forecast two-day patterns have lower accuracy than forecasting 1 day ahead, which means that there is a greater risk, leading to greater uncertainty. Similarly, in the forecast 3, 5, 7 and 10 days ahead, the pure ML methods forecast three-day patterns have lower accuracy than forecasting 1 day and 2 days ahead, which means that it may suffer continuous losses in the future. The line chart in **Fig 5** shows the predicted averages for all the segment patterns.

**Fig 6:**
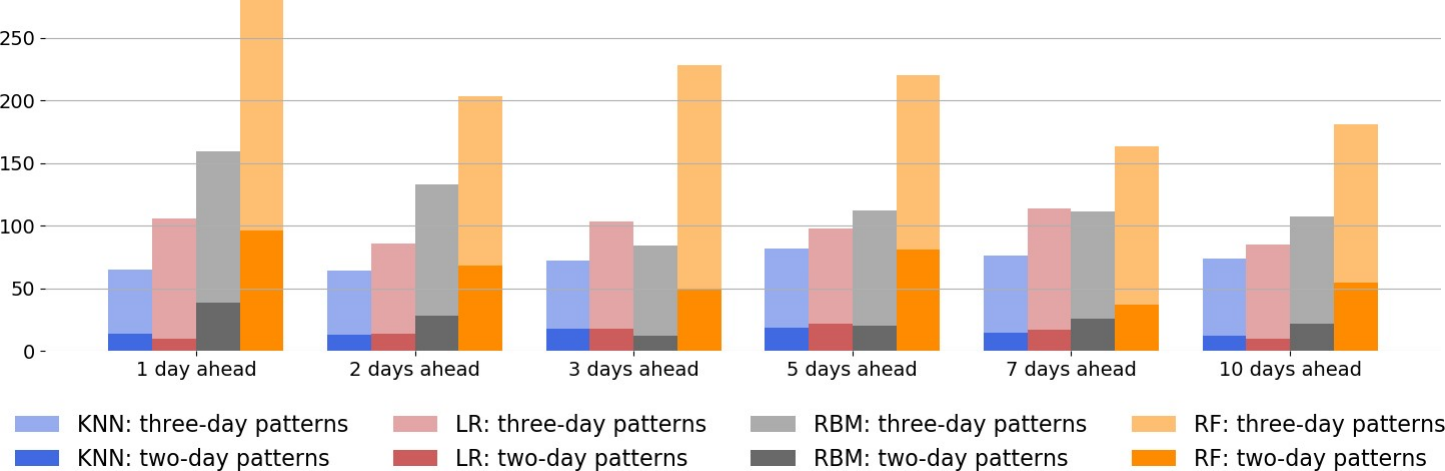
The number of the four machine learning methods supporting highest prediction accuracy.

For different candlestick patterns, four machine models have different prediction effects. Four machine learning models are used to make predictions separately for each pattern. The machine learning model with the highest prediction accuracy corresponding to each pattern will be recorded. **Fig 6** shows the number of machine learning methods whose prediction accuracy exceeds the threshold. Taking the two-day candlestick patterns which have 169 combinations in theory to predict one day ahead as an example, 159 patterns have a prediction accuracy rate that exceeds our threshold, thus supporting 1,299,028 rows of data. From the bottom half of **Fig 6**, we can see that 14 patterns perform well using the KNN prediction method, 10 patterns make the best predictions by using the LR method, 39 patterns are supported by the RBM model, and 96 models make the best predictions by using the RF method. Taking the three-day candlestick patterns which have 2,197 combinations in theory to predict one day ahead as an example, the prediction accuracy of 451 patterns exceeded our threshold and supported 665,243 rows of data. From the upper half of **Fig 6**, we can see that 51 patterns perform well using the KNN prediction method, LR supports 96 models, 120 patterns make the best predictions by using the RBM method, and RF supports 184 patterns. RF outperforms LR, KNN and RBM in two-day and three-day-pattern forecasting. Except for forecasting 5 days ahead, the number of patterns that exceeds the accuracy threshold value decreases significantly as the forecast period becomes longer based on two-days pattern forecasting. The number of patterns to meet the conditions for forecasting 1, 2, 3, 5, 7, and 10 days ahead are 159, 123, 98, 142, 95, and 99 respectively. In terms of the three-day patterns, the number of patterns in forecasting 1 day ahead is significantly higher than in forecasting other periods. The number of three-day patterns to meet the conditions for forecasting 1, 2, 3, 5, 7, and 10 days ahead are 451, 363, 389, 370, 369, and 348 respectively. Regardless of two-day patterns or three-day patterns, the number of selecting patterns for forecasting 1 day ahead is significantly higher than forecasting other periods, which means that it is possible to obtain better performance in forecasting 1 day than in other periods.

### 3.3 Investment strategy result

**Fig 7:**
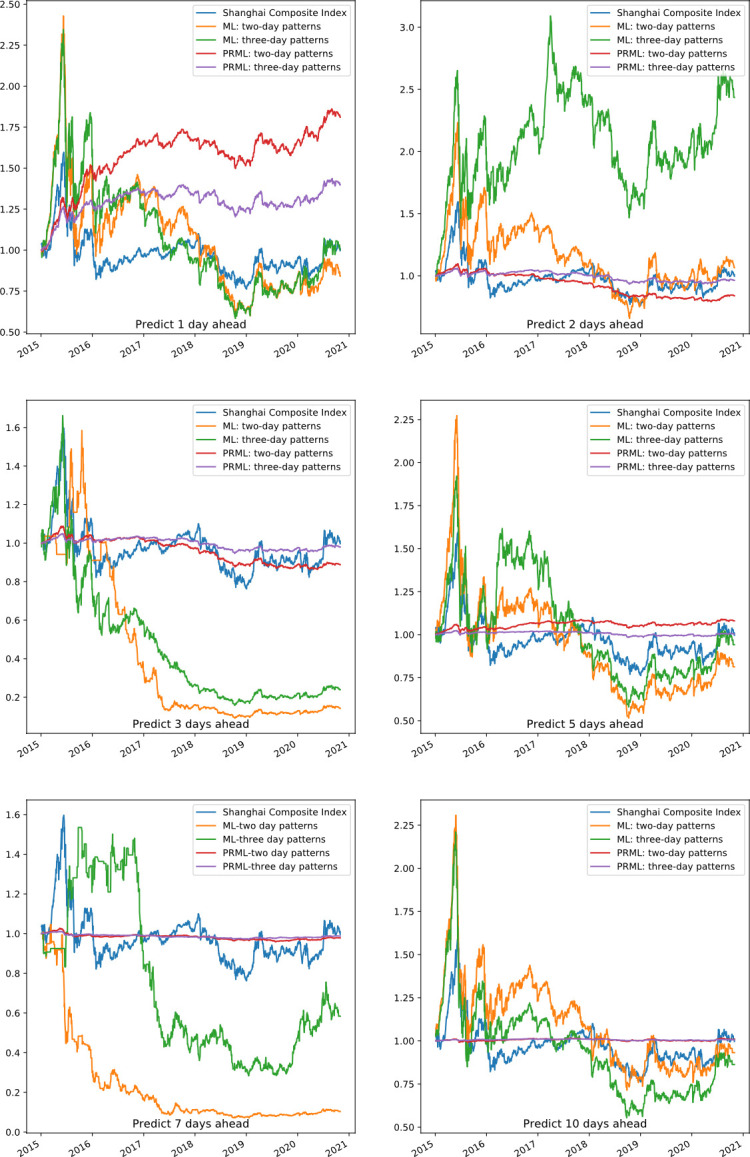
Portfolio forecasting performance of 1, 2, 3, 5, 7, 10 days ahead: PRML vs. ML.

Based on the PRML prediction framework of this paper, we conducted an investment validation in the forecasting sets. The cumulative return performance of PRML and pure ML, including forecasting 1, 2, 3, 5, 7, and 10 days ahead, is shown as **Fig 7**. The Shanghai Composite Index during the same period was used as a benchmark, as shown by the blue line in the figure. The performance of PRML with respect to two-day patterns and three-day patterns is better than pure ML when forecasting 1 day ahead and shows more stability when forecasting 2, 3,5,7 and 10 days ahead. For the two-day patterns, the pure ML models predict and give investment recommendations for 13*13 patterns every day. For the three-day patterns, the pure ML models predict 13*13*13 patterns and give investment recommendations every day. However, PRML only gives investment recommendations for the patterns whose prediction accuracy exceeds the threshold. Pure ML methods predict and give investment recommendations for each stock every day, while PRML invests according to the performance of different patterns, and the average number of stocks invested per day is relatively small. This can be seen from the number of selected patterns in **Fig 6** and the number of theoretical combinations. Lower forecast accuracy and frequent transactions may lead to a significant decline in earnings, as we can see from the forecast results of 3 and 7 days ahead in **Fig 7**. Pure ML methods have a better effect in predicting two days in the three-day patterns, which is consistent with the higher accuracy in **Fig 5**.

The finance performance of PRML and ML when predicting one day ahead is shown as **Table 3**. The average annual return of ML3, PRML2 and PRML3 show that the previous patterns are profitable. For both the two-day patterns and the three-day patterns, the financial performance of PRML is better than that of ML. With a maximum of 10.75% annual returns, the two-day patterns based on the PRML model are more profitable than the market. During the same period, two-day patterns using pure ML have a large drawdown of 75.45%, and our portfolio drawdown using PRML is smaller, thus indicating that the proposed model has less risk. In the one-day forecasting scenario, the two-day patterns are better than the three-day patterns.

**Table 3:** Finance performance of forecasting one day ahead: PRML vs. ML.

	SH Index	ML2	PRML2	ML3	PRML3
Average Annual Return	-0.66%	-2.89%	10.75%	0.41%	5.90%
Max Drawdown	-52.28%	-75.45%	-13.81%	-75.18%	-14.05%
Annual Sharpe Ratio	-0.19	-0.25	0.17	-0.28	-0.2
Information Ratio	0	0.41	2.38	0.38	2.08
Standard Deviation	1.48	2.09	0.49	2.11	0.42

* SH Index represents the Shanghai Composite Index, ML2 represents using two-day patterns without detail and pure ML to forecast, PRML2 represents using two-patterns and PRML to forecast, ML3 represents using three-day patterns without detail and pure ML to forecast, PRML3 represents using three-day patterns and PRML to forecast. Risk-free uses the yield to maturity of China’s government bonds in the past five years, with a value of 3.08%.

Then, 5 more detailed investment strategies are constructed based on PRML. First, 5 different strategy pools are generated based on machine learning results and investment strategy. Next, the prediction effects of different periods from 1 day to 10 days are examined. For each day of data in the prediction set, we use the corresponding machine learning method according to the pattern rule pool to forecast. If the prediction result is long, a buy operation is performed. Then, the result with the actual situation is compared and the average daily return is calculated with equal weight.

**Fig 8:**
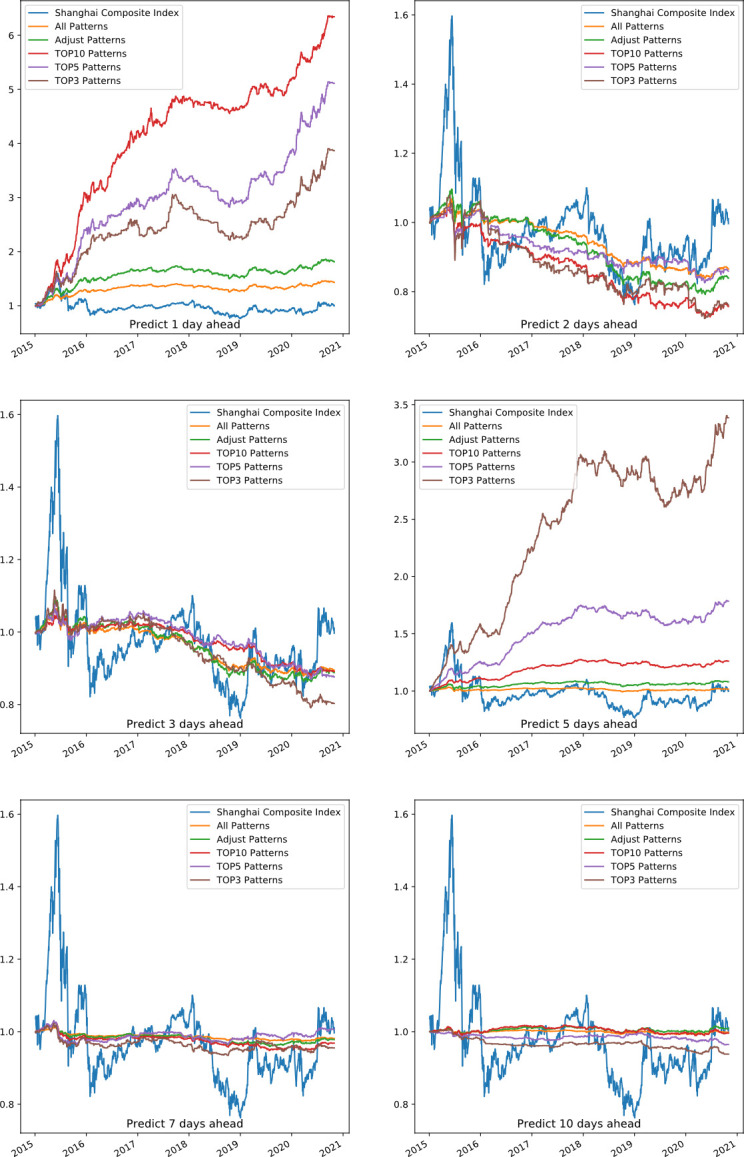
Portfolio return performance of two-day patterns predicting 1, 2, 3, 5, 7, 10 days ahead.

**Fig 8** shows the average portfolio return of two-day candlestick patterns, including forecasting 1, 2, 3, 5, 7, and 10 days ahead. *All* patterns indicate that we construct the portfolio according to all the patterns in the pattern rule pool. *Adjust* patterns show that we exclude the patterns in the baseline candlestick pattern pool that appear fewer than *1000* times in the machine learning set. The *TOP10* patterns indicate that we use the 10 most accurate candlestick patterns, *TOP5* means that we use only the 5 most accurate candlestick patterns, and *TOP3* indicates that we use the top 3 most accurate patterns to invest. It seems that the two-day combination patterns have a certain prediction effect for 1 and 5 days. This is consistent with the higher number of selected patterns in forecasting 1 day and 5 days ahead than 2, 3, 7 and 10 days ahead in **Fig 6**. In the case of two-day patterns forecasting 5 days ahead, the performance difference among *TOP3*, *TOP5 and TOP10* is obvious, indicating that its return is significantly affected by a certain pattern. Of the two-day pattern’s prediction effect for six different periods, the 1-day prediction effect is the best. The very short-term forecasts may be related to China’s emerging stock markets.

The finance performance of two-day candlestick patterns in predicting one day ahead is shown in **Table 4**. The average annual return of TOP3, TOP5 and TOP10 shows that the previous patterns are profitable. With a maximum annual return of 36.73% returns, five different portfolios based on the PRML model are more profitable than the market. During the same period, the market has a large drawdown of 52.28%, and our portfolio drawdown is smaller, thus indicating that the proposed model has less risk. The All and Adjust patterns show that using many patterns can reduce risk, but the corresponding profit will be reduced.

**Table 4:** Finance performance of two-day patterns predicting one day ahead.

	SH Index	All	Adjust	TOP10	TOP5	TOP3
Average Annual Return	-0.66%	6.35%	10.75%	36.73%	33.76%	26.17%
Max Drawdown	-52.28%	-10.90%	-13.81%	-16.43%	-15.34%	-27.62%
Annual Sharpe Ratio	-0.19	-0.08	0.17	0.81	0.99	0.62
Information Ratio	0	1.94	2.38	2.37	2.02	2.26
Standard Deviation	1.48	0.34	0.49	0.78	0.84	0.96

* All represents a portfolio constructed by all patterns whose training accuracy is higher than 55%, Adjust represents a portfolio constructed by the patterns whose training accuracy is higher than 55% and whose support number is higher than 1000, TOP10 represents a portfolio constructed by the top 10 training accuracy patterns, TOP5 represents a portfolio constructed by the top 5 training accuracy patterns, and TOP3 represents a portfolio constructed by the top 3 training accuracy patterns.

**Fig 9:**
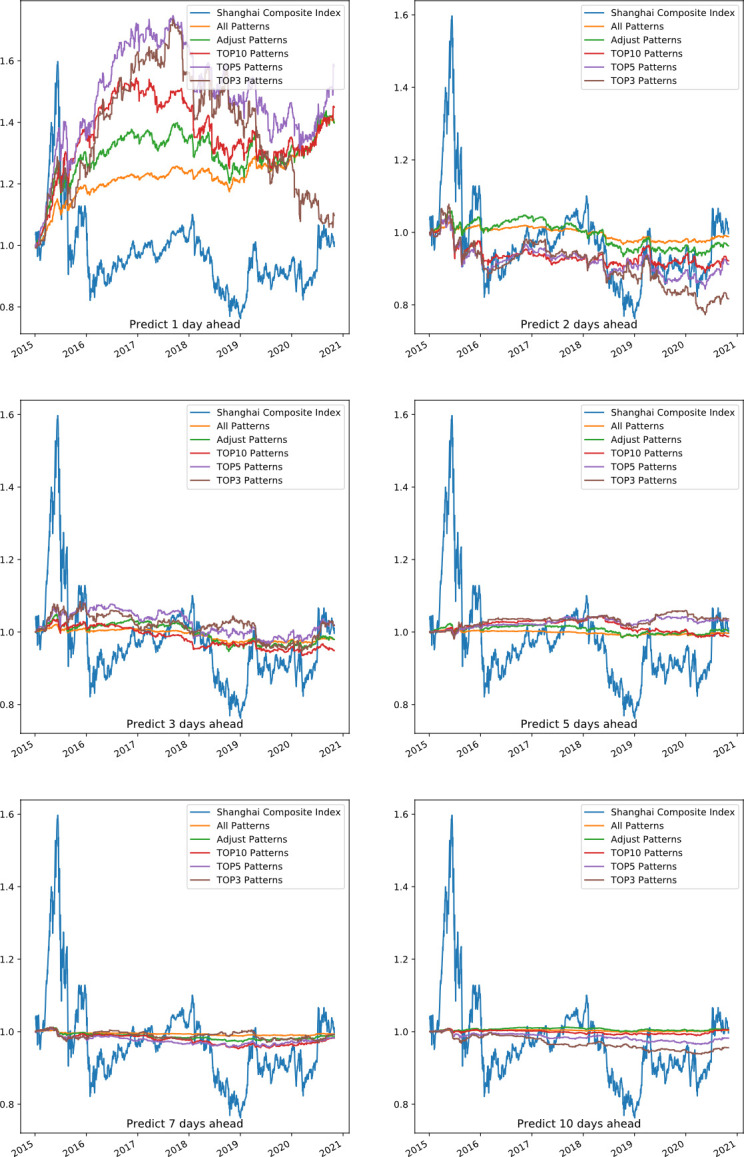
Portfolio return performance of three-day patterns predicting 1, 2, 3, 5, 7, 10 days ahead.

The portfolio average return of three-day patterns for forecasting 1, 2, 3, 5, 7, and 10 days ahead is shown as **Fig 9**. It shows that the three-day combination patterns have certain prediction effects only for 1 day. Among the prediction effect for six different periods, the one-day prediction effect is the best. This effect may be related to short-term market volatility.

**Table 5** shows the finance performance of three-day patterns to predict one day ahead. The average annual returns of the five strategies show that the previous patterns are profitable when they predict one day ahead. With a maximum annual return of 8.29%, five different portfolios based on the PRML model are more profitable than the market. During the same period, the market has a large drawdown of 52.28%, and our portfolio drawdown is smaller, thus indicating that the proposed model has less risk. The *All* and *Adjust* patterns show that using many patterns can reduce risk, but the corresponding profit will be reduced.

**Table 5:** Finance performance of three-day patterns predicting one day ahead.

	SH Index	All	Adjust	TOP10	TOP5	TOP3
Average Annual Return	-0.66%	5.95%	5.90%	6.62%	8.29%	1.74%
Max Drawdown	-52.28%	-6.63%	-14.05%	-19.10%	-24.90%	-39.00%
Annual Sharpe Ratio	-0.19	0.10	-0.2	-0.21	0.03	-0.22
Information Ratio	0	1.53	2.08	2.10	2.32	1.64
Standard Deviation	1.48	0.23	0.42	0.59	0.74	0.88

In the *All* strategy, although the three-day candlestick combination patterns have a lower return than do the two-day candlestick combination patterns, the max drawdown of the three-day candlestick combination patterns has also decreased and has greater stability. Portfolios constructed by the top 10 three-day candlestick patterns have not obtained a return larger than the return obtained by the top 5 portfolios; however, more patterns can bring greater benefits in the two-day candlestick patterns because there is a fewer supporting number of three-day patterns in the training set and the machine learning model is prone to overfitting. However, adding more three-day combination patterns can effectively increase stability.

Although we have used all the stock data of Chinese listed companies for 15 years, the training data corresponding to each pattern of the two-day patterns and the three-day patterns are different. Especially the three-day patterns which have as many as 2,197 combinations, the data of each pattern of the three-day patterns is still relatively small, which will easily cause the over-fitting of machine learning, leading to obvious differences in the prediction effects which are shown in Tables 4 and 5.

### 3.4 Prediction model dependence testing

In recent years, new developments in deep learning have allowed for multiple levels of abstraction. The deep learning models have performed well in speech recognition, text processing, etc. Following Krauss et al. (2017) and Yu et al. (2020), two popular deep learning models are applied to verify the dependence of the pattern recognition framework [22, 43]. The MLP and LSTM forecasting models are used in two-day patterns and three-day patterns to predict one day ahead. Following Patel et al. (2015) and Zhou et al. (2019), *Accuracy* and *F-measure* are used for screening and identifying patterns, and the qualified patterns will be automatically put into the strategy pool [3, 20].

**Fig 10:**
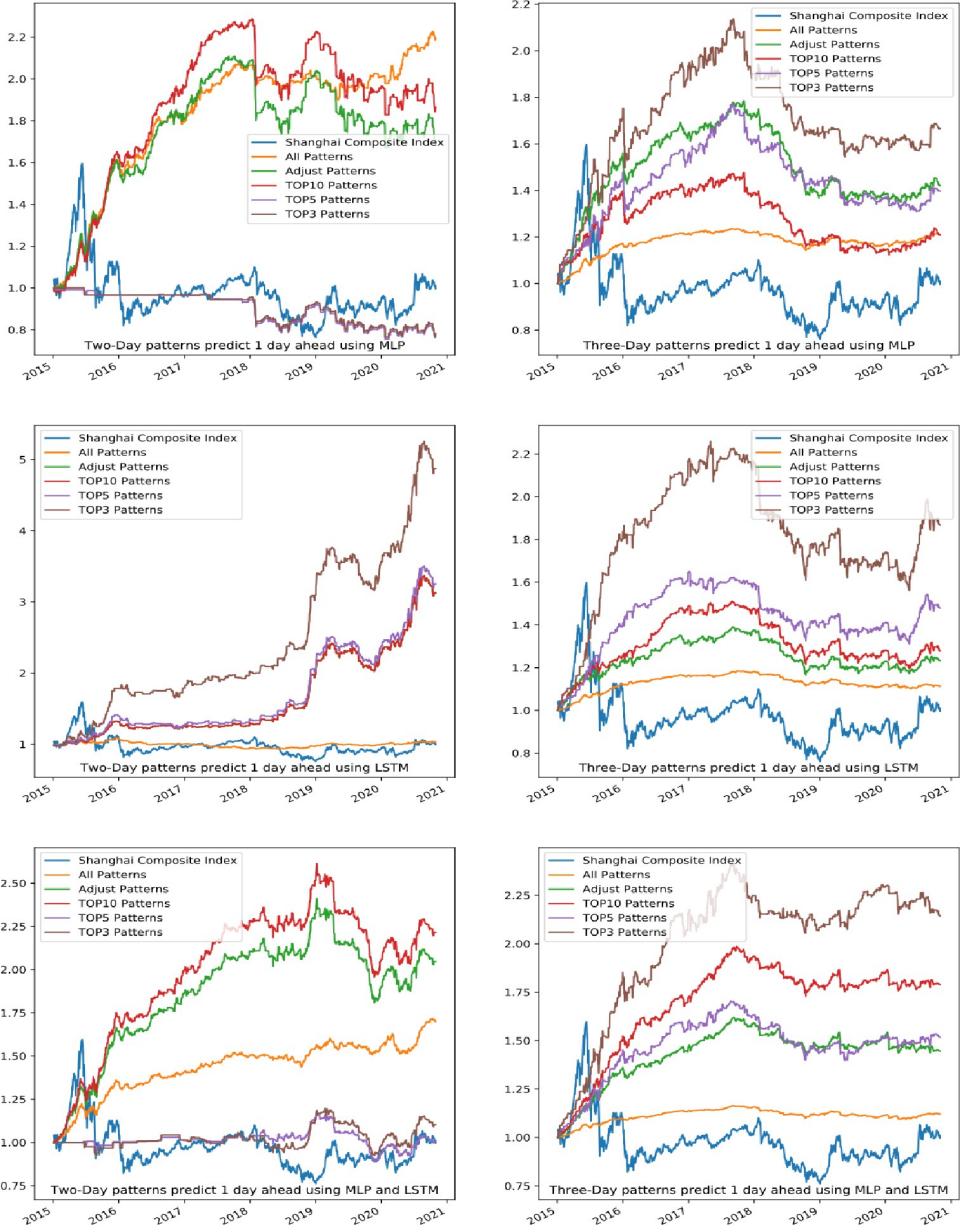
One day ahead forecasting performance of MLP and LSTM.

The portfolio return performance of MLP and LSTM is shown in **Fig 10**. The performance of the Shanghai Composite Index during the same period was used as a benchmark, which is indicated by the blue line in the figure. The figure shows that MLP and LSTM have obviously prediction effects in terms of one-day prediction. Applying other machine learning model methods to pattern recognition can also obtain excess returns.

The best strategy’s financial performance when using two-day and three-day patterns for one-day predictions is shown in **Table 6.** Among the strategies, with a maximum of 21.83% annual returns and 16.26% max drawdown, the TOP10 strategy using LSTM seems to obtain the best performance. The best-performing patterns during the training period are not profitable when using MLP to make one day predictions in two-day patterns; i.e., the stability is poor. Similar to the four traditional machine learning methods used in the previous PRML, MLP and LSTM are still profitable and have good prediction effects.

**Table 6:** One day ahead performance of MLP and LSTM.

	SH Index	Two-day predictions one day ahead			Three-day predictions one day ahead		
		TOP10 (MLP)	TOP10 (LSTM)	TOP10 (MLP+LSTM)	TOP3 (MLP)	TOP3 (LSTM)	TOP3 (MLP+LSTM)
Average Annual Return	-0.66%	11.43%	21.83%	14.72%	9.16%	11.34%	14.01%
Max Drawdown	-52.28%	-20.03%	-16.26%	-25.10%	-27.70%	-30.91%	-14.87%
Annual Sharpe Ratio	-0.19	0.04	0.75	0.21	-0.42	-0.14	0.04
Information Ratio	0	2.16	1.00	2.26	2.51	2.49	2.49
Standard Deviation	1.48	0.64	0.75	0.57	0.78	0.76	0.75

### 3.5 Further analysis

The performance of the PRML model on sliding window data is also tested. **Table 7** shows the performance of two-day patterns predicting one day ahead. It can be seen from the table that our forecasting model still has a good forecasting effect on four different data intervals, and the strategies are all profitable with a smaller retracement rate than the index.

**Table 7:** Finance performance of sliding windows in two-day patterns predicting one day ahead.

Different training and testing sets	Benchmark	SH Index	All	Adjust	TOP10	TOP5	TOP3
Training: Jan 1,2001-Dec 31,2015	Average Annual Return	-0.46%	10.44%	3.72%	3.97%	7.42%	9.78%
Testing: Jan 1,2016-Oct 30,2020	Max Drawdown	-30.73%	-9.16%	-17.61%	-17.32%	-23.48%	-28.49%
	Standard Deviation	1.19	0.27	0.45	0.51	0.73	0.96
Training: Jan 1,2002-Dec 31,2016	Average Annual Return	0.73%	12.19%	1.19%	1.12%	4.36%	3.55%
Testing: Jan 1,2017-Oct 30,2020	Max Drawdown	-30.73%	-9.87%	-18.94%	-21.44%	-21.30%	-5.99%
	Standard Deviation	1.11	0.27	0.46	0.53	0.73	0.29
Training: Jan 1,2003-Dec 31,2017	Average Annual Return	-1.32%	25.16%	11.65%	13.06%	33.58%	12.39%
Testing: Jan 1,2018-Oct 30,2020	Max Drawdown	-30.73%	-3.10%	-14.64%	-16.27%	-22.14%	-30.26%
	Standard Deviation	1.25	0.28	0.58	0.65	1.03	1.32
Training: Jan 1,2004-Dec 31,2018	Average Annual Return	15.80%	45.04%	44.83%	51.92%	21.79%	0.59%
Testing: Jan 1,2019-Oct 30,2020	Max Drawdown	-18.66%	-1.67%	-3.84%	-6.92%	-11.11%	-3.79%
	Standard Deviation	1.25	0.28	0.46	0.58	0.65	0.35

Finally, the effects of stock transaction costs, whose importance was emphasized by Park and Irwin [44] are examined. Caginalp and Laurent, and, Bessembinder and Chan note that the largest cost in the U.S. stock market is the bid-ask spread, which ranges from 0.1% to 0.39% [4, 8]. In the Chinese stock market, broker commissions range from 0.015% to 0.3%, and the stamp duty is 0.1% when selling shares. Therefore, we tested the two-day candlestick patterns’ one-day-ahead predictions with a total transaction cost of 0.2%.

**Fig 11:**
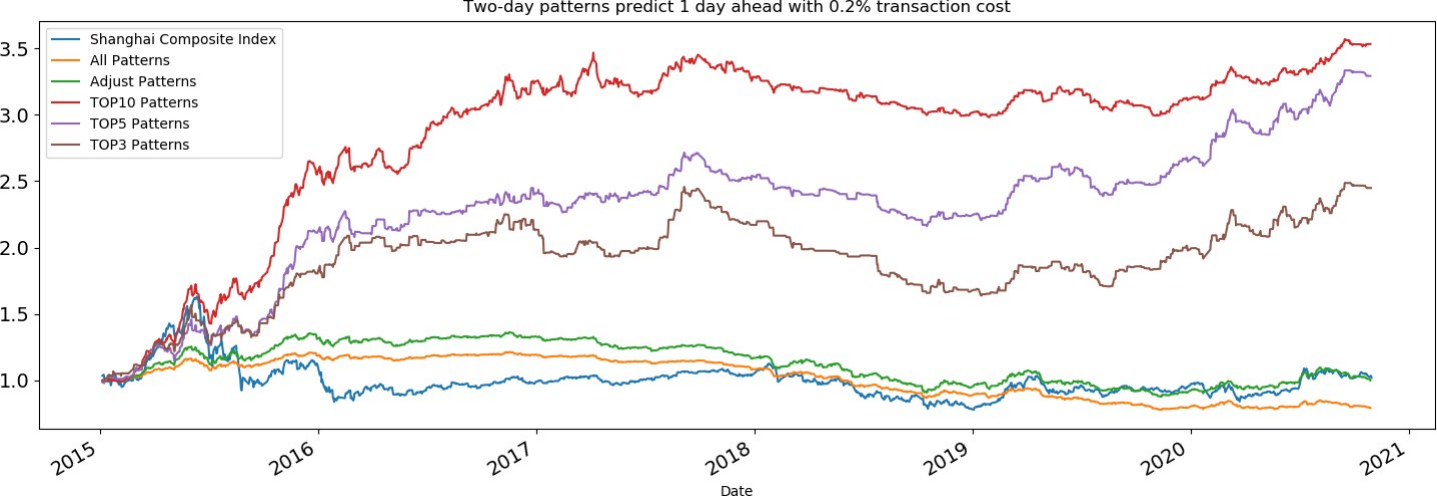
Portfolio return of two-day predictions one day ahead with 0.2% transaction cost.

The validity of the prediction results is shown as **Fig 11**. Although the returns have decreased, the results are not qualitatively changed even when a higher transaction cost is considered. The finance performance of two-day candlestick patterns in predicting one day ahead with 0.2% transaction cost is shown in **Table 8**. With a maximum average annual return of 24.45%, four different portfolios based on the PRML model investment strategy are all more profitable than the market. Additionally, the max drawdown of our portfolio is smaller than the max drawdown of the market during the same period.

**Table 8:** Finance performance of two-day predictions one day ahead with 0.2% transaction cost.

	SH Index	All	Adjust	TOP10	TOP5	TOP3
Average Annual Return	-0.66%	-3.92%	0.01%	24.45%	23.02%	16.65%
Max Drawdown	-52.28%	-35.85%	-35.66%	-17.29%	-20.38%	-33.31%
Annual Sharpe Ratio	-0.19	-1.37	-0.71	0.32	0.50	0.25
Information Ratio	0	0.16	0.86	2.68	2.31	2.49
Standard Deviation	1.48	0.41	0.62	0.74	0.84	0.96

## 4. Conclusion

Forecasting the direction of the daily changes of stocks is an essential yet challenging task. The newest data mining and artificial intelligence methods can be used to improve the effectiveness of financial market forecasting. This paper attempts to develop PRML, a pattern recognition model that uses machine learning methods to improve stock trading decisions. Different time windows from one to ten days are used to detect the prediction effect at different periods. Empirical results show that the two-day candlestick patterns and three-day candlestick patterns have the best prediction effect when forecasting one day ahead. In general, the results do not qualitatively change even when a 0.2% transaction cost is considered. Two other popular machine learning models are used to test the dependence of the prediction model. The MLP and LSTM forecasting models also perform well when predicting one day ahead. The results show that an investment strategy constructed according to the PRML model can be profitable.

This study contributes to the literature in four ways: First, a candlestick pattern recognition model based on machine learning methods is proposed. The prediction results obtained by this model are more accurate than those obtained by purely using machine learning methods. Four machine learning methods—LR, KNN, RBM, and RF—are applied to all possible different combinations of daily patterns in the pattern recognition process. We incorporate the shape, location and nine commonly used technical indicators as features into each machine learning model to improve the accuracy of predictions.

Second, we divide the data set into two parts—the machine learning set and the prediction set—and use the trained parameters to perform prediction tests on new unknown data in the experimental stage. Conventional machine learning methods generally divide the data set only into a training set and a testing set. We added a prediction set to test whether the identified candlestick patterns still have a prediction effect on unknown data. We split the data set into the following two parts during the experimental testing phase of China’s stock market: the machine learning data set from Jan 1, 2000 to Dec 31, 2014 and the prediction set from Jan 1, 2015 to Oct 30, 2020.

Third, this paper examines the effects of predicting at different periods. Based on the two-day and three-day candlestick patterns identified by PRML, an investment strategy was constructed to dynamically build a daily investment portfolio. The average number of stocks invested in the portfolio is significantly less than that of pure ML methods. Compared with the pure ML methods, the PRML can effectively improve the accuracy of prediction, thereby further reducing the risk of uncertainty. For the number of patterns that exceed the accuracy threshold, the 1 day ahead forecasting is significantly higher than other forecasting periods. The strategy results also show that we can obtain the best performance when predicting one day ahead. These very short-term forecasting effects may be related to the characteristic of China’s emerging stock markets. Compared to the -0.66% annual return of the market, all the identified two-day candlestick patterns yield an average annual return of 6.35%, and all the eligible three-day candlestick patterns have a 5.95% average annual return over the same period. Relative to the market drawdown, the max drawdown of the two-day candlestick pattern is 10.90%, and the max drawdown of the three-day candlestick pattern is only 6.63%.

Finally, five different strategy pools were used to form five comparable strategies to build daily portfolios dynamically. Five strategies (including *TOP3*, *TOP5*, and *TOP10*, which have the highest accuracy in the machine learning set), *All* patterns with an accuracy rate of more than 55%, and *Adjust* patterns that remove the candlestick patterns that occur fewer than 1000 times are used to dynamically build a daily portfolio. The results show that more two-day candlestick patterns can be profitable. After filtering, the two-day candlestick patterns have the best prediction effect when forecasting one day ahead and using the *TOP10* strategy, which, in this case, obtains an average annual return, an annual Sharpe ratio, and an information ratio as high as 36.73%, 0.81, and 2.37, respectively. With a maximum annual return of 8.29% from the *TOP5* strategy, three-day candlestick patterns after screening also present a profitable effect when forecasting one day ahead, thus showing more stable characteristics. During the same period, the market has a significant drawdown of 52.28%, and the max drawdowns of our portfolios are all less than 40%, thus indicating that the proposed model has less risk than the market.

## 5. Limitations and future work

Although we have used all the stock data of Chinese listed companies from 2000 to 2014 for learning, the number of each combination pattern is still relatively small. We hope to identify the candlestick patterns that have a certain frequency in the market. We did not identify more complex candlestick patterns in the empirical stage because more complex patterns require larger data sets. A more careful hyperparameter optimization of different machine learning methods, especially deep learning models, may still need to be considered in future research to get better prediction performance.

In future work, we will consider researching candlestick patterns in more mature markets. We also plan to utilize additional predictive factors, such as the newest deep learning methods and more technical indicators, to improve forecasting results.

## Supporting information

S1 Data (ZIP)

S2 Data (ZIP)
